# Polycomb Factor PHF19 Controls Cell Growth and Differentiation Toward Erythroid Pathway in Chronic Myeloid Leukemia Cells

**DOI:** 10.3389/fcell.2021.655201

**Published:** 2021-04-29

**Authors:** Marc García-Montolio, Cecilia Ballaré, Enrique Blanco, Arantxa Gutiérrez, Sergi Aranda, Antonio Gómez, Chung H. Kok, David T. Yeung, Timothy P. Hughes, Pedro Vizán, Luciano Di Croce

**Affiliations:** ^1^Epigenetics Events in Cancer Laboratory, Centre for Genomic Regulation (CRG), Barcelona Institute of Science and Technology, Barcelona, Spain; ^2^Rheumatology Department, Rheumatology Research Group, Vall d’Hebron Research Institute, Barcelona, Spain; ^3^Precision Medicine Theme, South Australian Health and Medical Research Institute (SAHMRI), Adelaide, SA, Australia; ^4^Adelaide Medical School, Faculty of Health and Medical Sciences, University of Adelaide, Adelaide, SA, Australia; ^5^Universitat Pompeu Fabra (UPF), Barcelona, Spain; ^6^ICREA, Barcelona, Spain

**Keywords:** chronic myeloid leukemia, polycomb, PHF19, epigenetics, erythroid differentiation

## Abstract

Polycomb group (PcG) of proteins are a group of highly conserved epigenetic regulators involved in many biological functions, such as embryonic development, cell proliferation, and adult stem cell determination. PHD finger protein 19 (PHF19) is an associated factor of Polycomb repressor complex 2 (PRC2), often upregulated in human cancers. In particular, myeloid leukemia cell lines show increased levels of PHF19, yet little is known about its function. Here, we have characterized the role of PHF19 in myeloid leukemia cells. We demonstrated that PHF19 depletion decreases cell proliferation and promotes chronic myeloid leukemia (CML) differentiation. Mechanistically, we have shown how PHF19 regulates the proliferation of CML through a direct regulation of the cell cycle inhibitor p21. Furthermore, we observed that MTF2, a PHF19 homolog, partially compensates for PHF19 depletion in a subset of target genes, instructing specific erythroid differentiation. Taken together, our results show that PHF19 is a key transcriptional regulator for cell fate determination and could be a potential therapeutic target for myeloid leukemia treatment.

## Introduction

Cell fate decisions rely on the precise control of specific transcription programs, which are governed by multiple layers of regulation. Among them, the epigenetic status of genes and their regulatory regions are paramount, not only because of their direct impact on expression but also for its reversible nature that allows a progressive fine-tuning control of expression along differentiation. The Polycomb group of proteins is one of the most important players in epigenetic regulation. They form multimeric complexes in the nucleus that associate with and modify the chromatin landscape. The Polycomb repressor complex 2 (PRC2) catalyzes the trimethylation of lysine 27 on the histone H3 N-terminal tail (H3K27me3), which is associated with chromatin compaction and gene repression (Schuettengruber et al., 2017). Apart from the PRC2 core components (EZH2, SUZ12, and EED), several sub-stoichiometric accessory factors have been described to regulate PRC2 genomic localization and function (Vizan et al., 2015; Laugesen et al., 2019). We and others have previously characterized the role of PHF19 (a mammalian homolog of the *Drosophila melanogaster* Polycomb-like, PCL) in embryonic stem cells (Ballare et al., 2012; Brien et al., 2012; Hunkapiller et al., 2012) and, more recently, in the hematopoietic system (Vizan et al., 2020). In both cases, in normal conditions, *PHF19* expression is reduced during differentiation, indicating its potential role in maintaining stem/progenitor characteristics.

Cancer could be considered as a failure to achieve or maintain unambiguous differentiated cell fates. Leukemia is the generic name given to several types of cancers, characterized for the accumulation of undifferentiated cells called blasts, either in the blood system, in the bone marrow, or in the lymph nodes (Islam, 1992). Myeloid leukemias are a group of leukemias where the cancerous cells derive from the myeloid precursors, which in normal conditions give rise to erythrocytes, platelets, monocytes, or granulocytes. Chronic myeloid leukemia (CML) is a clonal disorder of the hematopoietic system that accounts for 15% of the newly diagnosed leukemias in adults (Jabbour and Kantarjian, 2020) and is characterized by the unregulated growth of non-functional erythroid cells and platelets in the peripheral blood, as well as marked myeloid hyperplasia in the bone marrow (Sawyers, 1999). CML was the first type of leukemia associated with a chromosomal translocation: the Philadelphia chromosome [t(9;22) (q34;q11.2)]. This translocation results in a fusion protein called BCR-ABL, which generates an anomalous tyrosine kinase activity that not only leads to the speeding of cell division but also promotes genomic instability, making the cell more susceptible to accumulate extra genetic mutations (Hehlmann et al., 2007). The specific tyrosine kinase inhibitor imatinib is nowadays the most common treatment in CML. However, almost 30% of patients develop resistance to imatinib (Kok et al., 2019). In this context, new treatments are required, and epigenetic factors have lately gained attention (Machova Polakova et al., 2013).

In the last few years, several reports have addressed the role of PHF19 in different types of cancers, including melanoma (Ghislin et al., 2012), prostate (Jain et al., 2020), glioblastoma (Deng et al., 2018), and also hematopoietic malignancies of the lymphoid branch, such as multiple myeloma (Ren et al., 2019; Mason et al., 2020), as well as its specific role in preventing T cell exhaustion (Ji et al., 2019). However, its role in myeloid leukemias has not been elucidated. Interestingly, from those reports, it is becoming clear that, although PHF19 could be considered as an enhancer of cell proliferation, the consequences of its depletion could be hazardous since it would lead to the generation of slow-growing undifferentiated cells that ultimately may increase the malignant properties of tumoral cells (Ghislin et al., 2012; Jain et al., 2020). Therefore, the role of PHF19 in myeloid leukemias, which are a clear example of the accumulation of undifferentiated progenitors, needs to be further investigated. In this study, we demonstrated not only the antiproliferative effects of targeting PHF19 in myeloid leukemias but also how its reduction leads to specific differentiation of CML cells toward the erythroid pathway.

## Results

### PHF19 Expression Regulates Chronic Myeloid Leukemia Performance

**FIGURE 1:**
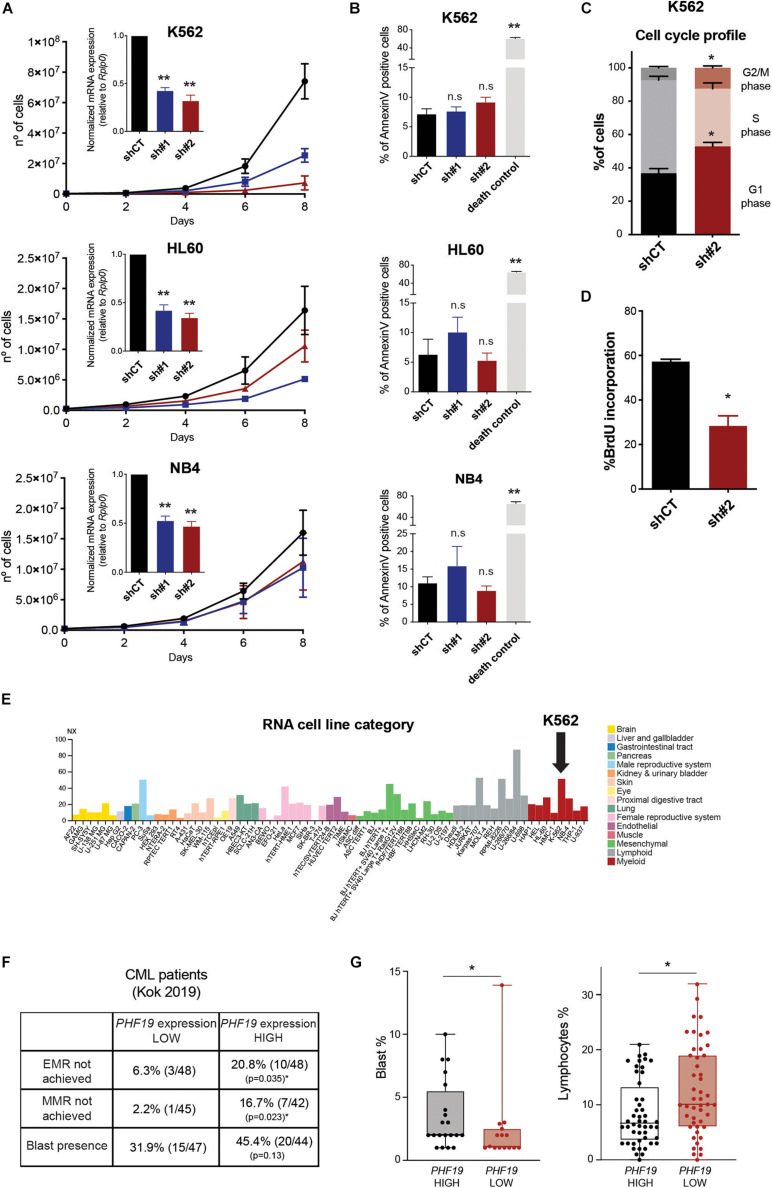
**(A)** Accumulative growth curve of cells infected with an empty short hairpin RNA (shRNA, *shCT*) and two shRNAs against *PHF19* (*sh#1* and *sh#2*). The *X*-axis shows the days after puromycin selection (see section “Materials and Methods”). *Inner panels*: levels of *PHF19* expression measured by qPCR relative to the housekeeping gene *Rplp0* and normalized by shCT expression 2 days after selection. Mean (*n* > 3 for all cases) + SEM. **(B)** Apoptosis assessed by the percentage of annexin V-positive cells in shCT-, sh# 1-, and sh#2-infected cells. Cell death control: non-infected cells under puromycin selection for 3 days. Mean (*n* = 2) + SEM. **(C)** Percentage of cells in the G1, S, and G2/M phases after propidium iodide (PI) staining 4 days after selection. Mean (*n* = 4) + SEM. **(D)** Percentage of BrdU-positive cells in shCT- and sh#2-infected K562 cells 4 days after selection. Mean (*n* = 3) + SEM. **(E)** Expression levels of *PHF19* in a panel of cell lines obtained from the Protein Atlas Database (cited in the main text). **(F)** Percentage and absolute number of patients that did not achieve EMR (early molecular response) or MMR (multiple molecular response) and presented blasts at peripheral blood samples, grouped by *PHF19* levels. **(G)** Boxplots and individual values of the percentage of blasts in patients where they could be detected (*left panel*) and percentage of lymphocytes (*right panel*) in peripheral blood samples. *⁢p<0.05;p**<0.01.

Three human myeloid-related leukemia cell lines (one of chronic myeloid leukemia origin: K562; two from acute promyelocytic leukemia: HL60 and NB4) were depleted for the long isoform of *PHF19* using two different short hairpin RNAs (shRNA) (Figure 1A, inner panels). In all cellular models, we observed a decrease in cell growth (Figure 1A), which was not due to an increase in apoptosis induction (Figure 1B). K562 was the most affected cell line, particularly when using sh*PHF19*#2. Of note is that K562 cell growth reduction upon EZH2 depletion using shRNAs has been reported, but accompanied by an increased apoptotic rate (Xie et al., 2016; Liu et al., 2017). Hence, we decided to profile the cell cycle by propidium iodide staining. Interestingly, a shortening in the S phase could be detected, together with an enrichment in both the G1 and G2/M phases upon *PHF19* depletion (Figure 1C and Supplementary Figure 1A): G1 increase from 36.8 ± 5.6% to 52.9 ± 4.9% and G2/M from 7.3 ± 1.5% to 12.5 ± 2.4%. This slow cell growth was confirmed by a reduction in the incorporation of the synthetic nucleoside BrdU (Figure 1D and Supplementary Figure 1B).

Among the human cancer cell lines reported in the Protein Atlas Database (Figure 1E; Uhlen et al., 2017)^1^, myeloid malignancies showed a high level of expression for *PHF19*, with K562 falling into the top three among all cell lines analyzed. We then wondered whether PHF19 plays a significant role in CML. Thus, we analyzed the expression data from blood samples in a cohort of almost 100 CML patients who were followed after imatinib treatment (Kok et al., 2019). When segregated by *PHF19* expression (50% highest vs. 50% lowest expressing patients), a differential response to treatment was observed: a high expression of *PHF19* correlated with poorer clinical outcomes (Figure 1F), i.e., a higher proportion of the patients with high *PHF19* expression failed to achieve early molecular response (EMR) or multiple molecular response (MMR) after the treatment. Although not statistically significant, the presence of blasts was also proportionately increased in the *PHF19* high group of patients. Moreover, when the percentage of blasts for positive samples was examined, it was significantly greater in the *PHF19* high fraction, which was also accompanied with a decrease in the percentage of total lymphocytes in the blood taking into account the whole cohort (Figure 1G). In this regard, the combination of PHF19 depletion in K562 cells with a lower concentration of imatinib causes a similar effect on cell growth (Supplementary Figure 1C). Taken together, these results pointed to a specific role of PHF19 in CML.

### *PHF19* Expression Controls the Differentiation Transcription Program Toward Erythroid

**FIGURE 2:**
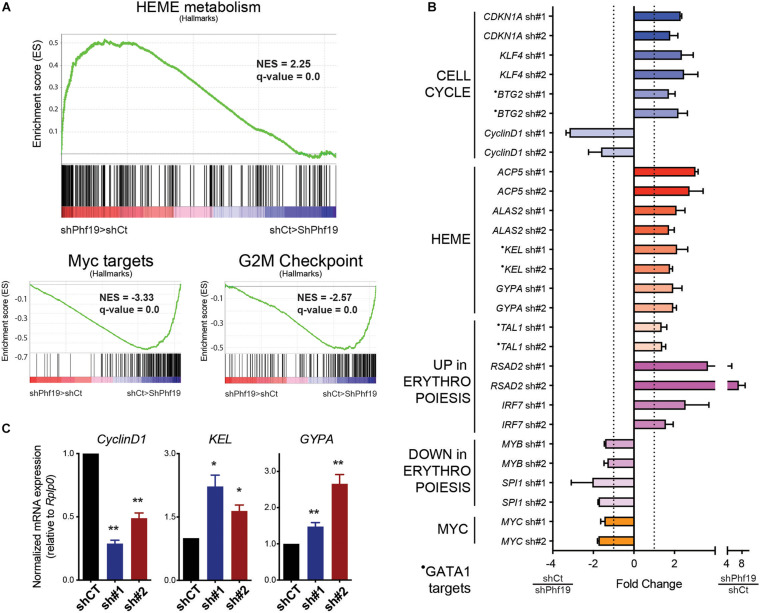
**(A)** Gene set enrichment analysis (GSEA) showing positive enrichment in the *PHF19*-depleted transcriptome for *Heme metabolism* and negative enrichment for *Myc targets* and *G2/M checkpoint* (hallmarks). **(B)** Normalized fold change expression (using reads per kilobase of transcript per million mapped reads, RPKMs) for the two short hairpin RNAs (shRNAs) against PHF19 (sh#1 and sh#2) with respect to cells infected with shCT (empty shRNA) in relevant genes from two independent RNA-seq experiments. Mean + SEM. **(C)** Expression of cyclin D1 (*CCND1*), *KEL*, and *GYPA* in sh#1 and sh#2 K562 cells, measured by qPCR relative to the housekeeping gene *RPLP0* and normalized by shCT expression. Mean (*n* > 3 in all cases) + SEM. *⁢p<0.05;p**<0.01.

To further characterize the role of PHF19 in K562 cells, we performed transcriptomic analysis after RNA isolation and massive sequencing (RNA-seq) in control cells and upon *PHF19* depletion. Both the shRNAs applied in Figure 1A were used for two independent replicates, and the expression differences were ranked using DESeq2 (Love et al., 2014) for gene set enrichment analysis (GSEA) (Subramanian et al., 2005). Unbiased GSEA pre-ranked analysis using hallmark signatures (Liberzon et al., 2015) rendered *Heme metabolism* as the top enriched category in depleted cells (Figure 2A). Interestingly, the K562 cell line could be potentially differentiated toward erythroid cell fate. Among the enriched categories in the control cells, we found *Myc targets*, whose decrease has also been clearly documented for erythroid differentiation (Jayapal et al., 2010), and *G2/M checkpoint* (Figure 2A), which correlated with the cell cycle arrest observed in Figures 1A–D.

We further analyzed our data by selecting the top 300 upregulated and downregulated genes according to DESeq2 ranking and performed Gene Ontology (GO) using the Enrichr web tool (Kuleshov et al., 2016). Among the categories we found in the upregulated group were *regulation of G1/S transition of mitotic cell cycle* (*GO:2000045*) (GO_Biological_Process, *p* = 0.031), *negative regulation of mitotic cell cycle* (*GO:0045930*) (GO_Biological_Process, *p* = 0.035), *Heme Biosynthesis WP561* (WikiPathways, *p* = 0.0075), *GATA1 CHEA* (ENCODE_and_ChEA Consensus_TFs_from_ChIP-X, *p* = 0.019), and *CD71* + *EarlyErythroid* (Human_Gene_Atlas, *p* = 0.02). Conversely, *MYC ENCODE* (ENCODE_and_ChEA Consensus_TFs_from_ChIP-X, *p* = 0.00000025), and *CD34*^+^ (Human_Gene_Atlas, *p* = 0.00012) were found in the downregulated group. Examples of the upregulated and downregulated genes are depicted in Figure 2B, and some were validated in independent experiments (Figure 2C). Cell cycle categories were found in the upregulated group of genes, in agreement with the data reported in Figures 1A–D and with the GSEA analysis (Figure 2A). Of note is that GATA1 transcriptional activity is required for erythroid differentiation (Tanimura et al., 2016). Contrary to *Myc*, which is directly downregulated upon *PHF19* depletion, *GATA1* expression was not directly affected upon *PHF19* knockdown, but many of its targets were found upregulated, mimicking its effects. The complementary upregulation on genes assigned to CD71^+^ cells and the downregulation of CD34^+^, which is a marker of undifferentiated cells (Orfao et al., 2019), are consistent with the role of PHF19 maintenance of undifferentiated cell status (Ballare et al., 2012). Among the upregulated genes, we also noted *Acp5*, whose expression is required for fetal erythroid differentiation (Ying et al., 2014); *Gypa*, which translates in the surface marker CD235a widely used for functional characterization of erythroid commitment (Fajtova et al., 2013; Li et al., 2014); as well as other markers [both upregulated (*Rsad2* and *Irf7*) and downregulated (*Myb* and *SpiI*)] used for erythroid cell determination (Liang et al., 2015). In sum, transcriptomic profiling indicated that PHF19 plays an important role in K562 cell fate determination.

### PHF19 Regulates p21 Expression in CML

**FIGURE 3:**
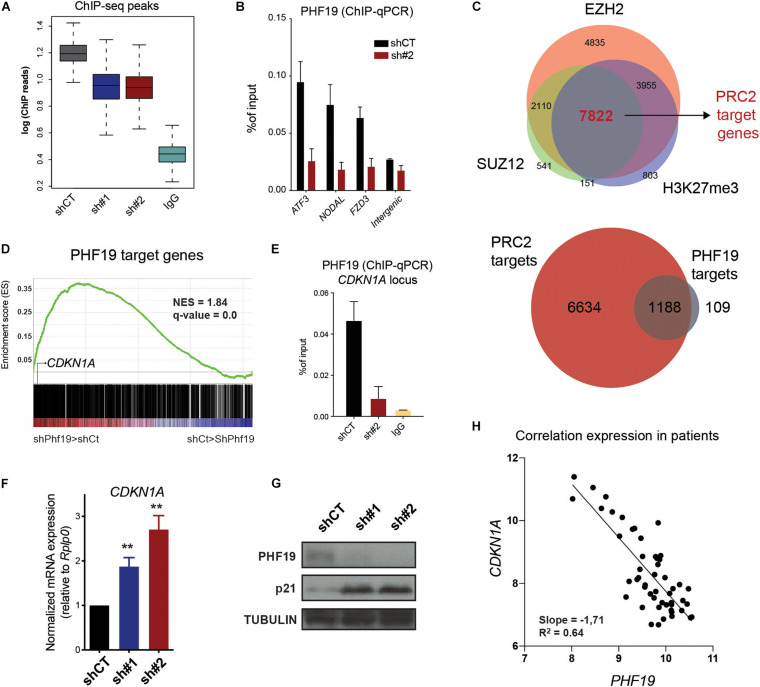
**(A)** Boxplot of the total number of reads in PHF19 chromatin immunoprecipitation sequencing (ChIP-seq) peaks. **(B)** PHF19 ChIP enrichment (with respect to the input) measured by qPCR of several genes and intergenic region as a negative control for the cells infected with an empty short hairpin RNA (shRNA, *shCT*) and a shRNA against PHF19 (sh#2). Mean (*n* > 2) + SEM. **(C)** Venn diagrams showing the intersection between the EZH2, SUZ12, and H3K27me3 target genes (*top*) and between these common targets and PHF19 target genes (*bottom*). **(D)** Gene set enrichment analysis (GSEA) showing positive enrichment in the *PHF19*-depleted transcriptome for the PHF19 target genes. **(E)** PHF19 ChIP enrichment (with respect to the input) measured by qPCR of the *CDKN1A* gene for shCT- and sh#2-infected K562 cells, as well as ChIP using IgG in shCT cells. **(F)** Expression of *CDKN1A* measured in sh#1- and sh#2-infected K562 cells, measured by qPCR relative to the housekeeping gene *RPLP0* and normalized by the expression of shCT-infected cells. Mean (*n* = 8) + SEM. **(G)** Western blot for PHF19 and p21 of shCT-, sh# 1-, and sh#2-infected K562 cells 5 days after selection; TUBULIN is used as a loading control. **(H)** Scatter plot showing the correlation of *PHF19* and *CDKN1A* microarray expression values measured from all bone marrow precursors of chronic myeloid leukemia (CML) patients (cited in the main text). ^∗∗^*p* < 0.01.

Since PHF19 is an epigenetic factor associated with PRC2, we hypothesize that its chromatin profile could offer valuable clues to understanding its role in regulating cell cycle and differentiation. To determine PHF19 localization on chromatin, we performed chromatin immunoprecipitation followed by massive sequencing (ChIP-seq) in control and *PHF19*-depleted K562 cells, together with IgG as the ChIP control (Figure 3A). We identified 2,328 PHF19 peaks genome-wide, significantly enriched in the promoter regions (Supplementary Figure 2A), corresponding to 1,297 target genes (Figure 3A). Several target genes (depicted in Supplementary Figure 2B) were further validated by ChIP-quantitative PCR (qPCR) in independent experiments (Figure 3B). Then, we defined bona fide PRC2 targets by intersecting EZH2, SUZ12, and H3K27me3 target genes from the K562 ChIP-seq data released by the ENCODE project (Consortium, 2012; Figure 3C, top panel). More than 90% of the PHF19 target genes were shared with PRC2 (Figure 3C, bottom panel), indicating a very low impact of PHF19 outside its canonical function as associated sub-stoichiometric Polycomb factor. We then reasoned that, as part of PRC2, PHF19 may have a general role as a transcriptional repressor. Indeed, the GSEA of the PHF19 gene targets showed significant enrichment in the upregulated genes upon depletion (Figure 3D). Among those, *CDKN1A* (p21) called our attention and was further validated by ChIP-qPCR around its transcription start site (Figure 3E). Moreover, we observed an increased expression at the RNA and protein levels in the *PHF19* knockdown conditions (Figures 3F,G). We thus wondered whether the PHF19-dependent control of p21 was a consistent mechanism for cell cycle modulation at the bone marrow of CML patients, where most cycling cells are found. Remarkably, using published data (Abraham et al., 2016), we corroborated the anticorrelation between *Cdkn1a* and *PHF19* expressions (Figure 3H).

### Specific MTF2 Compensation Upon PHF19 Reduction Defines PRC2 Role in K562

**FIGURE 4:**
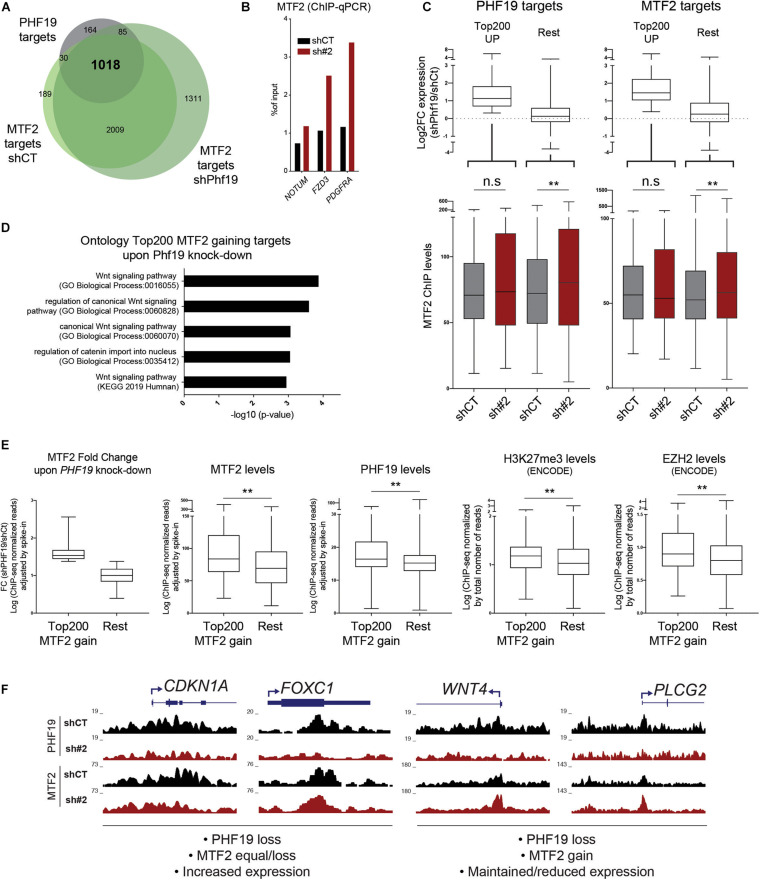
**(A)** Venn diagram showing the intersection between the PHF19 gene targets and the MTF2 gene targets for cells infected with an empty short hairpin RNA (shRNA, *shCT*) and a shRNA against PHF19 (*sh#2*). **(B)** MTF2 chromatin immunoprecipitation (ChIP) enrichment (with respect to the input) measured by qPCR of several genes and intergenic region as a negative control in shCT- and sh#2-infected K562 cells. **(C)** *Top*: Fold change of sh*PHF19* vs. shCT (Log2) of the top 200 upregulated vs. the rest of the PHF19 (*left*) and MTF2 (*right*) gene targets. *Bottom*: Levels (maximum peak high) of the MTF2 ChIP signal in shCT- and sh#2-infected K562 cells of the top 200 upregulated vs. the rest of the PHF19 (*left*) and MTF2 (*right*) gene targets. **(D)** Gene ontology of the top 200 MTF2 targets that increase their levels upon *PHF19* depletion. **(E)** Characterization of the top 200 MTF2 targets that increase their levels upon *PHF19* depletion versus the rest of the MTF2 levels. From *left* to *right*: Fold change of MTF2 gain and levels (maximum peak high) of MTF2, PHF19, H3K27me3, and EZH2. **(F)** Representative screenshots modified from the UCSC Genome Browser. ^∗∗^*p* < 0.01.

Overexpression of p21 may not be sufficient to induce differentiation toward erythroid fate in K562. Recently, the depletion of the PHF19 homolog MTF2 has been linked to erythropoiesis in a knockout mouse model (Rothberg et al., 2018). These two factors may be competing for interaction with the PRC2 core components. Although the *MTF2* levels did not change upon *PHF19* depletion, we wondered whether MTF2 occupancy would be affected by changes in PHF19 expression. Thus, we performed MTF2 ChIP-seq in *PHF19*-depleted cells and found that: (i) most of the PHF19 targets overlapped with the MTF2 targets (Figure 4A, and Supplementary Figure 2C), indicating redundancy not only for the complex formation but also for the same genomic regions; (ii) MTF2 binding was more spread across the genome; and (iii) there was an increase in the number of target genes upon *PHF19* depletion (Figure 4A). In fact, this increase was also noticeable in the ChIP-seq signal strength upon *PHF19* knockdown, which we validated for several genomic targets (Figure 4B and Supplementary Figure 2D). However, the observed general derepression of PHF19 target genes upon depletion of *PHF19* seemed incompatible with MTF2 compensation. Certainly, the expression changes are not homogeneous (Figure 3D), and we hypothesized that, although MTF2 might be able to compensate the lack of PHF19 in many target genes, there is a subset of genes where the reduction of PHF19 directly leads to the upregulation of expression. To investigate this possibility, we selected the top 200 ranked upregulated PHF19 target genes and analyzed changes in the MTF2 levels upon *PHF19* knockdown. We corroborated that the fold change expression of these selected genes was consistent with the DESeq2 ranking (Figure 4C, top left panel). Interestingly, the MTF2 levels did not significantly change for those targets that were highly derepressed. In contrast, a significant increase of the MTF2 signal was observed in the rest of the targets (Figure 4C, bottom left panel). Moreover, we reasoned that the absence of PHF19 would allow an increased interaction of MTF2 with the PRC2 core components, which would affect its function beyond PHF19 targets. Therefore, we performed the same analysis for the top 200 MTF2 target genes upregulated upon *PHF19* depletion. Similarly, the derepressed genes did not show an increase in MTF2 levels, as observed and expected for the rest of the target genes (Figure 4C, right panels).

These results suggested a distinct behavior of MTF2 compensation after *PHF19* reduction. To explore what caused this difference irrespective of the expression, we selected the top 200 MTF2 targets that showed the stronger increase of MTF2 occupancy upon *PHF19* depletion (Figure 4E, left panel). Firstly, we studied the GO of these selected targets, and strikingly, the top two categories obtained by GO were the *Wnt signaling pathway* and *regulation of canonical Wnt signaling*. Wnt-related categories were also found significantly enriched when pathways (Kyoto Encyclopedia of Genes and Genomes, KEGG) were queried (Figure 4D). Of note is that, in the GO of the 200 lower PHF19 target genes ranked by expression upon *PHF19* depletion, we could also detect Wnt-related categories: *Regulation of Wnt signaling pathway* (GO_Biological_Process, *p* = 0.00079) and *Wnt signaling pathway* (KEGG, *p* = 0.0052). This is concordant with the reported MTF2 role in hematopoiesis since repression of the Wnt signaling pathway by MTF2 instructs erythroid differentiation (Rothberg et al., 2018). To gain insights into why MTF2 is increasingly deposited in a subset of targets, we studied their epigenetic status in control conditions: as depicted in Figure 4E, the ChIP-seq levels of MTF2, PHF19, H3K27me3, and EZH2 were higher in the top 200 MTF2 target genes with respect to the rest, indicating they were significantly occupied by PRC2 prior to *PHF19* depletion. Finally, Figure 4F depicts representative examples of the chromatin profiles for PHF19 and MTF2 upon *PHF19* depletion. As could be observed, *CDKN1A* or *FOXC1* (transcriptionally upregulated upon *PHF19* depletion) did not display an MTF2 increase, contrary to what could be observed in the Wnt-related genes *WNT4* (transcriptomically unaffected) and *PLCG2* (transcriptionally downregulated).

### PHF19 Depletion Enhances Erythroid Differentiation While Impeding Megakaryocyte Cell Fate Induction

**FIGURE 5:**
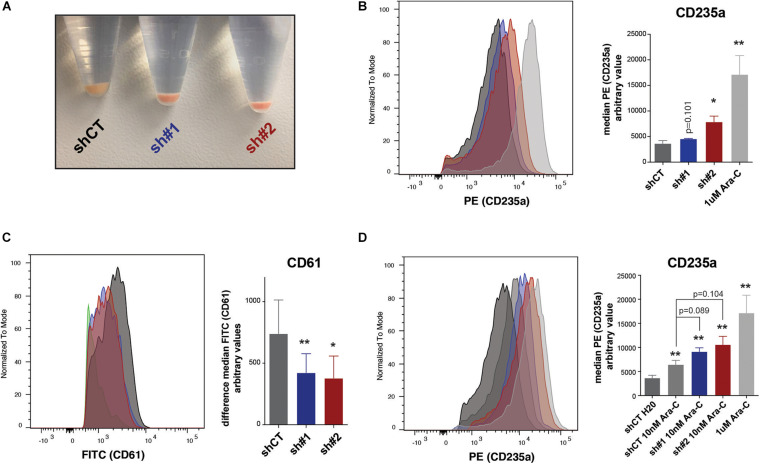
**(A)** Picture of pelleted K562 infected with an empty short hairpin RNA (shRNA, *shCT*) and two shRNAs against PHF19 (*sh*#1 and *sh#2*) 5 days after puromycin selection. **(B)** CD235a marker levels measured by flow cytometry in shCT-, sh# 1-, sh#2-infected cells 4 days after puromycin selection and 1 μM of Ara-C treatment in uninfected cells as a positive control. *Left*, representative plot of a single experiment. *Right*, bar plot of the mean of median cytometry values (shCt and sh#1, *n* = 4; sh#1 and 1 μM of Ara-C, *n* = 3) + SEM. **(C)** CD61 marker levels measured by flow cytometry in shCT-, sh# 1-, and sh#2-infected cells 4 days after puromycin selection and treated with 1 nM phorbol myristate acetate (PMA) for 24 h. *Left*, representative plot of a single experiment. *Right*, bar plot of the mean of median cytometry values (*n* = 3) + SEM. **(D)** CD235a marker levels measured by flow cytometry at 4 days in shCT-, sh# 1-, and sh#2-infected cells after puromycin selection and treated with 10 nM of Ara-C for 72 h, with 1 μM of Ara-C treatment in uninfected cells as a positive control. *Left*, representative plot of a single experiment. *Right*, bar plot of the mean of median cytometry values (shCt, 10 nM Ara-C; sh#1, 10 nM Ara-C, *n* = 24; sh#1, 10 nM Ara-C, and 1 μM of Ara-C, *n* = 3) + SEM. *⁢p<0.05;p**<0.01.

Throughout the experiments with shRNAs, we noticed that cell pellets from the *PHF19*-depleted cells acquired a pale reddish color (Figure 5A), possibly indicating a hasty production of hemoglobin. Therefore, we reasoned that, beyond epigenetic and transcription indications toward erythroid differentiation, K562 cells were already acquiring phenotypic erythroid characteristics. To assess this, we analyzed by flow cytometry the presence of the well-known erythroid precursor marker CD235a (Fajtova et al., 2013; Li et al., 2014) using cells treated with Ara-C (cytarabine) as a positive control (Zhang et al., 2007; Cai et al., 2014). Both shRNAs caused an increase of CD235a (Figure 5B). To corroborate that the effect on differentiation upon *PHF19* depletion is specific, we forced the differentiation of K562 cells into the megakaryocyte lineage by phorbol myristate acetate (PMA) treatment (Cai et al., 2014) after *PHF19* depletion and measured the megakaryocyte surface marker CD61 (Ogino et al., 2014). Indeed, the cells with reduced levels of *PHF19* were resistant to acquire megakaryocyte characteristics (Figure 5C). Finally, Ara-C is used as a treatment in CML and other leukemias^2^, and according to our results, reduction of the PHF19 levels could be proposed for cooperative CML treatments. To test this, we reduced a hundred times the concentration of Ara-C used as a positive control in Figure 5B to produce a more modest increase in the CD235a marker, and we measured differentiation and cell growth upon PHF19 depletion. Interestingly, Ara-C cell growth inhibition was enhanced by PHF19 depletion (Supplementary Figure 2E); more interestingly, it was able to further enhance the differentiation of K562 cells in the presence of low Ara-C-treated cells (Figure 5D).

## Discussion

Here, we have shown that reduced PHF19 levels in CML cells arrest the cell cycle and promote differentiation toward erythroid fate. The role of PRC2 in leukemia has been studied (Carlo et al., 2019), and in particular, the catalytic PRC2 core component EZH2 has been reported to be overexpressed in CML (Xie et al., 2016). Moreover, EZH2 inhibition reduces cell growth and sensitizes CML cells to tyrosine kinase inhibitors (Scott et al., 2016; Xie et al., 2016). Furthermore, in line with our results, it has been recently reported that the oncogene MYCN regulates EZH2 expression in CML cells, which leads to p21 repression, increasing proliferation and blocking differentiation (Liu et al., 2017). However, due to its pleiotropic functions, targeting the PRC2 core components may disrupt many cellular functions, and thus focusing on the sub-stoichiometric accessory factors, such as PHF19, may be an advantage. A fine example of this was our recent study on the role of PHF19 in normal mouse hematopoiesis: previous loss-of-function studies of PRC2 core components demonstrated their essential role in hematopoiesis (Xie et al., 2014; Lee et al., 2015; Yu et al., 2017), but the associated lethality had hampered an in-deep characterization of the transcriptional pathways or H3K27me3 (re)distribution in hematopoietic stem cells (HSCs). We generated a *Phf19* knockout mice, which resulted viable and then allowed us to unveil its role in controlling adult HSC dormancy (Vizan et al., 2020). Similarly, this study has allowed us to determine a very specific epigenetic mechanism by which high levels of PHF19 impede erythroid differentiation, which could have been impossible to evaluate by blocking the entire PRC2 activity.

To achieve differentiated characteristics, the cell cycle has to be arrested. The CML cell line K562 harbors the BCR-ABL fused tyrosine kinase, which activates the signaling cascades in charge of cell cycle regulation. At the same time, p53 is truncated and is not functional in these cells (Law et al., 1993), leaving p21 as one of the few factors able to avoid uncontrolled cell proliferation. We demonstrated that PHF19 occupies the p21 promoter, and its depletion coincides with p21 derepression, pointing to a plausible mechanism of how PHF19 overexpression impacts on cell growth. Surprisingly, a recent study has reported no effects on cell proliferation in K562 upon *PHF19* depletion (Ren et al., 2019). Nonetheless, we hypothesize that this may be due to different targeting and/or reduction efficiency. On the other hand, p21 overexpression may account for cell cycle arrest, but it might not be enough to induce the specific erythroid differentiation reported. The K562 cell line has been previously described as erythroleukemia (Chylicki et al., 2000), but it also has been largely known as a model for megakaryoblast differentiation (Alitalo, 1990). In fact, it has been previously shown that p21-induced cell cycle arrest favors megakaryocyte differentiation (Munoz-Alonso et al., 2005). Therefore, additional cellular and molecular mechanisms might be triggered upon *PHF19* depletion.

We noticed that, although the main function of PHF19 is transcriptional gene repression, a subset of the PHF19 target genes remain unaffected or are even more repressed upon its depletion. In this sense, a recent report has demonstrated that another PCL homolog, MTF2, is required for normal erythropoiesis (Rothberg et al., 2018). We have studied the occupancy of MTF2 in response to *PHF19* depletion, and we have observed that it differentially compensates the PHF19 loss in a subset of targets. Interestingly, the gain of MTF2 is more pronounced among those genes whose expressions are non-derepressed, including the Wnt pathway, which remarkably needs to remain repressed in order to achieve erythroid characteristics (Rothberg et al., 2018). Some of these genes reduce their expressions, but also a part of them remains unaffected, probably because their expression levels were already low to ensure further differentiation steps.

Which other chromatin or regulatory features determine where in the genome MTF2 is compensating for PHF19 reduction remains to be comprehensively elucidated. Worthy of note is our observation that those genes in which MTF2 did not compensate for PHF19 loss displayed reduced levels of the PRC2 components, which led us to hypothesize that repressive chromatin status is warranted specifically in a subset of genes to ensure cell differentiation. Therefore, we foresee a model where high levels of PHF19 in CML cancer cells would lead to small albeit enough accumulation in other genomic targets. This would induce a degree of unexpected gene repression, heightening the already disturbed normal differentiation. In other words, a gain in *PHF19* expression would intensify the resemblance to myeloid precursors, where cell fate has not been yet decided. In conclusion, this study confirms the necessity of maintaining tight control of epigenetic regulation to sustain proper adult stem cell differentiation as well as reinforces the possibility of using specific acquired epigenetic vulnerabilities for tumor differentiating therapies.

## Materials and Methods

### Cell Culture and Growth Curves

NB4, HL60, and K562 cells were cultured at 37°C and 5% of CO_2_ in RPMI medium supplemented with 10% fetal bovine serum. HEK293T cells were cultured at 37°CC and 5% CO_2_ in Dulbecco’s modified Eagle’s medium (DMEM) supplemented with 10% fetal bovine serum.

To monitor cell growth, leukemic cell lines were seeded at 2.5 × 10^5^ cells/ml for the NB4 and HL60 cell lines and 3 × 10^5^ cells/ml for the K562 cell line, then the cells were counted and seeded again every other day. Imatinib (Thermo Fisher Scientific) and Ara-C (Sigma) were added after puromycin selection and cell growth monitored for 6 days.

### Lentiviral Production and Infection

The PLKO.1 lentiviral system was used for the production of shRNAs against *PHF19*. The target sequences were AAGCTTCCATCCACATGTGTT for shRNA#1 and GCCACACATTTGAGAGCATCA for shRNA#2. Empty vector was used as the control (shCT). Viral particles were produced in HEK293T, which were plated at a density of 2 × 10^6^ cells in a p10 plate and transfected the following day by adding dropwise while vortexing a CaCl_2_–DNA solution (10 μg of pLKO.1 of plasmid, 6 μg of pCML-dR8.91, 5 μg of pCMV-VSGV, and 62 μl of 2 M CaCl_2_ in a final volume of 0.5 ml) previously incubated at room temperature (RT) for 15 min to an equal volume of HBS 2 × (HEPES-buffered saline solution, pH 7.05, 0.28 NaCl, 0.05 M HEPES, and 1.5 mM Na_2_HPO_4_). After cell incubation for 14–16 h, the transfection medium was replaced by fresh medium and the cells were incubated for 24 h. The medium with the lentiviral particles was harvested and filtered through a 45-μm filter. Then, fresh medium was added to the HEK293T and the media with lentiviral particles were harvested again the following day.

Two rounds of leukemic cell infection were performed on six-well plates according to the days the medium was harvested. Of the cells, 5 × 10^5^ were plated in 1 ml of medium, and then 1 ml of the medium with lentivirus was added. Cells were spinoculated (1,000 × *g*, 90 min, 32°C) in the presence of protamine sulfate (1 μg/ml). After the two rounds of infection, the cells were selected with 1 μg/ml of puromycin.

### Apoptosis, Cell Cycle, and BrdU Incorporation

The cell apoptosis assay was performed using violet annexin V/Dead Cell Apoptosis Kit (Invitrogen) according to the manufacturer’s protocol. Post-staining, the cells were analyzed by flow cytometry. For cell cycle, 1 × 10^6^ cells were washed with cold phosphate-buffered saline (PBS) and resuspended in 0.9 ml of EDTA-PBS (5 mM EDTA). Then, the cells were permeabilized by adding 2.1 ml of 100% cold ethanol dropwise while the mixture was softly shaken. The cells were kept at 4°C until the following day, when they were resuspended in propidium iodide (PI) staining buffer that contains 955 μl PBS + 30 μl of solution A (38 μl of 0.5 M sodium citrate + 562 μl of 500 μg/ml propidium iodide) + 2 μl of RNAse A (Thermo Fisher). The cells then were incubated at 37°C for 1 h and analyzed by flow cytometry. Cell cycle profiles were analyzed with the ModFit LT^TM^ software. For BrdU assay, the cells were treated with 10 μM of BrdU solution for 30 min and processed using the BrdU Flow Kit (BD Pharmingen) according to the manufacturer’s protocol. The percentage of BrdU-positive cells was analyzed by flow cytometry.

### Cell Differentiation by Surface Markers

The K562 cells either in normal conditions or after Ara-C (Sigma) or PMA (Sigma) treatment were rinsed twice with PBS and incubated for 45 min with conjugated antibodies against CD235a-PE (Invitrogen #12-9987-82) or CD61-FITC (eBioscience #11-0619-42) at 4°C, protected from light. After washing twice with cold PBS, the cells were suspended in PBS with DAPI to discard the non-viable cells and then analyzed by flow cytometry. Analysis was performed using FlowJo software.

### Western Blot

Four days after selection, infected (shCT, sh#1, and sh#2) cell pellets were washed twice with PBS and resuspended in hypotonic buffer (10 mM Tris–HCl, pH 7.4, 10 mM KCl, and 15 mM MgCl_2_) in the presence of protease and phosphatase inhibitors. After 10 min of incubation on ice, the resuspension was centrifuged (700 × *g*) for 5 min at 4°C. The pellet was resuspended in nuclear lysis buffer (300 mM NaCl, 50 mM HEPES, pH 7.5, 0.5% NP40, and 2.5 mM MgCl_2_) in the presence of protease and phosphatase inhibitors and benzonase (50 U/500 μl of buffer). The resuspension was centrifuged (16,000 × *g*) for 30 min at 4°C and the supernatant was considered the protein extract of the nuclear protein. Protein concentration was quantified by the Bradford assay (Bio-Rad). Seventy micrograms of protein was diluted in 5 × Laemmli buffer and heated for 5 min at 100°C. Protein samples were loaded in a NuPAGE^TM^ 4–12% Bis-Tris precast gel (Invitrogen). The proteins were transferred onto nitrocellulose membranes at 300 mA for 70 min at 4°C, blocked with 5% milk in TBS-Tween (10 mM Tris–HCl, pH 7.5, 100 mM NaCl, and 0.1% Tween-20) for 30 min at room temperature, and incubated at 4°C in TBS-Tween 5% milk overnight with the following primary antibodies: PHF19 (Cell Signaling #77271), p21 (Cell Signaling #2947), and TUBULIN (Abcam #7291). The following day, the membranes were washed with TBS-Tween followed by incubation of the secondary antibody conjugated to horseradish peroxidase (1:5,000) TBS-Tween for 1 h at room temperature. Then, the membranes were washed twice with TBS-Tween at room temperature. The proteins were then detected with enhanced chemiluminescence reagent (Pierce ECL Western Blotting Substrate, Thermo Scientific).

### Gene Expression

RNA was extracted with the RNeasy Mini Kit (Qiagen) according to the manufacturer’s protocol.

#### Expression by qPCR

cDNA was generated from 1 μg of RNA with the First Strand cDNA Synthesis Kit (Fermentas) according to the manufacturer’s instructions. Real-time PCR (qPCR) reactions were performed using the SYBR Green I PCR Master Mix (Roche) and the Roche LightCycler 480. The primers used were the following: *PHF19* Fw (CAGCAGAAAAGGCGAGTTTATAG), *PHF19* Rv (CTCCAGGCTGAGGTGAAGTC); *CCND1* Fw (GCCGA GAAGCTGTGCATC), *CCND1* Rv (CCACTTGAGCTTG TTCACCA); *KEL* Fw (ACCATGGGGAGACTGTCCT), *KEL* Rv (GGGCTTCCTACACATCACCT); *GYPA* Fw (CAA ACGGGACACATATGCAG), *GYPA* Rv (TCCAATAACACCAG CCATCA); *CDKN1A* Fw (CAGCTGCCGAAGTCAGTTCC), *CDKN1A* Rv (GTTCTGACATGGCGCCTCC).

#### RNA-Seq

RNA samples were quantified and the quality evaluated using Bioanalyzer. Libraries were prepared at the UPF/CRG Genomics Unit using 1 μg total RNA and sequenced using the Illumina HiSeq2000 sequencer. RNA-seq reads were mapped against the hg19 human genome assembly using TopHat (Trapnell et al., 2009) with the option –g 1 to discard those reads that could not be uniquely mapped in just one region. DESeq2 (Love et al., 2014) was run to quantify the expression of every annotated transcript using the RefSeq catalog of exons and to identify each set of differentially expressed genes. Reads per kilobase of transcript per million mapped reads (RPKMs) were used for boxplots and to calculate the fold change differences between conditions. GSEA of the pre-ranked lists of genes by DESeq2 stat value was performed with the GSEA software (Subramanian et al., 2005).

### Chromatin Immunoprecipitation and Analysis

Cells (25 × 10^6^) were harvested and washed twice with PBS and cross-linked in two steps. Firstly, the cells were resuspended in 10 ml of PBS 1 mM MgCl_2_ and 40 μl of ChIP Cross-link Gold (Diagenode) and incubated, shaking for 30 min at RT. Secondly, the cells were resuspended in 1% formaldehyde for 10 min at RT and fixation stopped by adding glycine to a final concentration of 0.125 M, then incubated for 5 min at RT. Then, the cells were washed twice with cold PBS. Chromatin preparation and ChIP experiments were performed with the ChIP-IT High Sensitivity Kit from Active Motif (#53040) according to the manufacturer’s instructions. ChIPs were performed using 5 μg/ChIP of the following antibodies: PHF19 (Cell Signaling #77271), MTF2 (ProteinTech 16208-1-AP), and control IgG (Abcam #ab172730). For spike-in control, an equal amount of *D. melanogaster* S2 cell chromatin was added to each ChIP reaction (0.1% of the K562 cell chromatin), together with 1 μg of an antibody against a *Drosophila*-specific histone variant, H2Av (Active Motif, #61686).

#### ChIP Quantification by qPCR

Real-time PCR (qPCR) reactions were performed using the SYBR Green I PCR Master Mix (Roche) and the Roche LightCycler 480. The primers used were the following: *ATF3* Fw (GTGGGTGGTCTGAGTGAGGT), *ATF3* Rv (CACAGTT TGGTAATTTGGGGTAG); *NODAL* Fw (GCGACTTCCTTAC TCGACCTC), *NODAL* Rv (CACAGTTTGGTAATTTGGGGT AG); *FZD3* Fw (AAAAGCACGTGCCATGAAT), *FZD3* Rv (CCTCCTTCATGGAGCCAGT); *CDKN1A* Fw (ATGTCATCC TCCTGATCTTTTCA), *CDKN1A* Rv (AGAATGAGTTGGCA CTCTCCAG); *NOTUM* Fw (CCGAGGCTGGGCTTATTT), *NOTUM* Rv (GGGAAGAAAAGGCGATGC); *PDGFRA* Fw (GGGGTGTCAGTTACAGAAGGTCT), *PDGFRA* Rv (CTGCCTGGATTAAAGTGTTAGGG); *INTERGENIC* Fw (ACAGGATAAAGTTGGCATAACCA); *INTERGENIC* Rv (CAACAAAACCGTTTGGAATACAT).

#### ChIP-Seq

For ChIP-seq experiments, library preparation was performed from 2–10 ng of precipitated chromatin at the UPF/CRG Genomics Unit. The libraries were sequenced using the Illumina HiSeq2000 sequencer. ChIP-seq reads containing spike-in were mapped against a synthetic genome constituted by human and fruit fly chromosomes (hg19 + dm3) using Bowtie with the option -m 1 to discard the reads that did not map uniquely to one region (Langmead et al., 2009). MACS was run with the default parameters, but with the shift size adjusted to 100 bp to perform the peak calling against the corresponding control sample (Zhang et al., 2008). In the PHF19 ChIP-seqs, only peaks with tags >70 were considered as positive. The genome distribution of each set of peaks was calculated by counting the number of peaks fitted on each class of region according to RefSeq annotations. Promoter is the region between 2.5 Kbp upstream and 2.5 Kbp downstream of the transcription start site (TSS). Genic regions correspond to the rest of the gene (the part that is not classified as promoter), and the rest of the genome is considered to be intergenic. Peaks that overlapped with more than one genomic feature were proportionally counted the same number of times. Each set of target genes was retrieved by matching the ChIP-seq peaks in the region 2.5 Kbp upstream of the TSS until the end of the transcripts as annotated in RefSeq. The signal strength or ChIP-seq level was calculated as the maximum high of peaks within the same region normalized by the fly spike-in number of reads of the same experiment. For EZH2, SUZ12, and H3K27me3 ENCODE data, raw reads and peaks were downloaded from GEO series GSE29611 (GSM1003576, GSM1003545, and GSM733658). The UCSC Genome Browser was used to generate the screenshots of each group of experiments along the manuscript (Kent et al., 2002).

### Data Availability

The datasets generated and analyzed for this study can be found in the National Center for Biotechnology Information Gene Expression Omnibus (Barrett et al., 2013) repository under the accession number GSE164804.

### Statistics

The number of replicates for each experiment is detailed in the corresponding figure legends or main text. For *PHF19* levels, apoptosis, cell cycle, BrdU incorporation, and qPCR expression data, paired *t* test was used. For lymphocyte counts and ChIP-seq levels, unpaired *t* test was used. For the patient data in Figure 1F, Fisher’s exact test was used. For blast counts in positive patient samples, the Mann–Whitney test was used. For the ratio of CD235a and CD61 levels, paired *t* test was used. Significance was set as ^∗^*p* < 0.05; ^∗∗^*p* < 0.01 throughout the study.

## Data Availability Statement

The datasets generated and analyzed for this study can be found in the National Center for Biotechnology Information Gene Expression Omnibus (Barrett et al., 2013) repository under the accession number GSE164804.

## Author Contributions

MG-M and CB planned and performed the experiments, and analyzed and interpreted the data. EB performed the bioinformatic analysis of genome-wide data, and analyzed and interpreted the results. ArG performed the experiments, and analyzed and interpreted the data. SA analyzed and interpreted the data. AnG performed analysis of patient data, and analyzed and interpreted the results. CK, DY, and TH provided patient data and interpreted the results. PV and LD conceived and planned the project, analyzed and interpreted the data, and wrote the manuscript, with the assistance and final approval of all authors. All authors contributed to the article and approved the submitted version.

## Conflict of Interest

The authors declare that the research was conducted in the absence of any commercial or financial relationships that could be construed as a potential conflict of interest.

## Footnotes

1 https://www.proteinatlas.org/ENSG00000119403-PHF19/cell

2 https://www.drugs.com/ppa/cytarabine-conventional.html

## Supplementary Material

Supplementary Figure 1 **(A)** Representative analysis of cell cycle phases in shCT and sh*PHF19*#2. **(B)** Representative analysis of BrdU incorporation analysis in shCT and sh*PHF19*#2. **(C)** Cell growth of cells infected with an shCT and shPHF19#2 in the absence or presence of 10 nM Ara-C for 6 days.

Supplementary Figure 2 **(A)** Genomic distribution of ChIP-seq peaks of PHF19. The spie-chart represents the genomic distribution of ChIP-seq peaks (outer circle) corrected by the whole-genome distribution of each gene genomic feature (indicated in the background circle distribution). **(B)** PHF19 ChIP-seq screenshots modified from UCSC genome browser of genes validated in Figure 3B. **(C)** Genomic distribution of ChIP-seq peaks of MTF2 (in shCT and sh*PHF19*#2). The spie-chart represents the distribution of peaks corrected by the genome-wide distribution of each gene genomic feature (indicated in the background circle distribution). **(D)** MTF2 ChIP-seq screenshots modified from UCSC genome browser of genes validated in Figure 4B. **(E)** Accumulative growth of cells infected with an shCT and sh*PHF19*#2 in the absence or presence of different doses of Imatinib for 6 days.
