# Supplementary figures and images for: Polycomb Factor PHF19 Controls Cell Growth and Differentiation Toward Erythroid Pathway in Chronic Myeloid Leukemia Cells
Source: Front Cell Dev Biol. 2021 Apr 29;9:655201. doi: 10.3389/fcell.2021.655201 (PMC8116664; doi:10.3389/fcell.2021.655201)

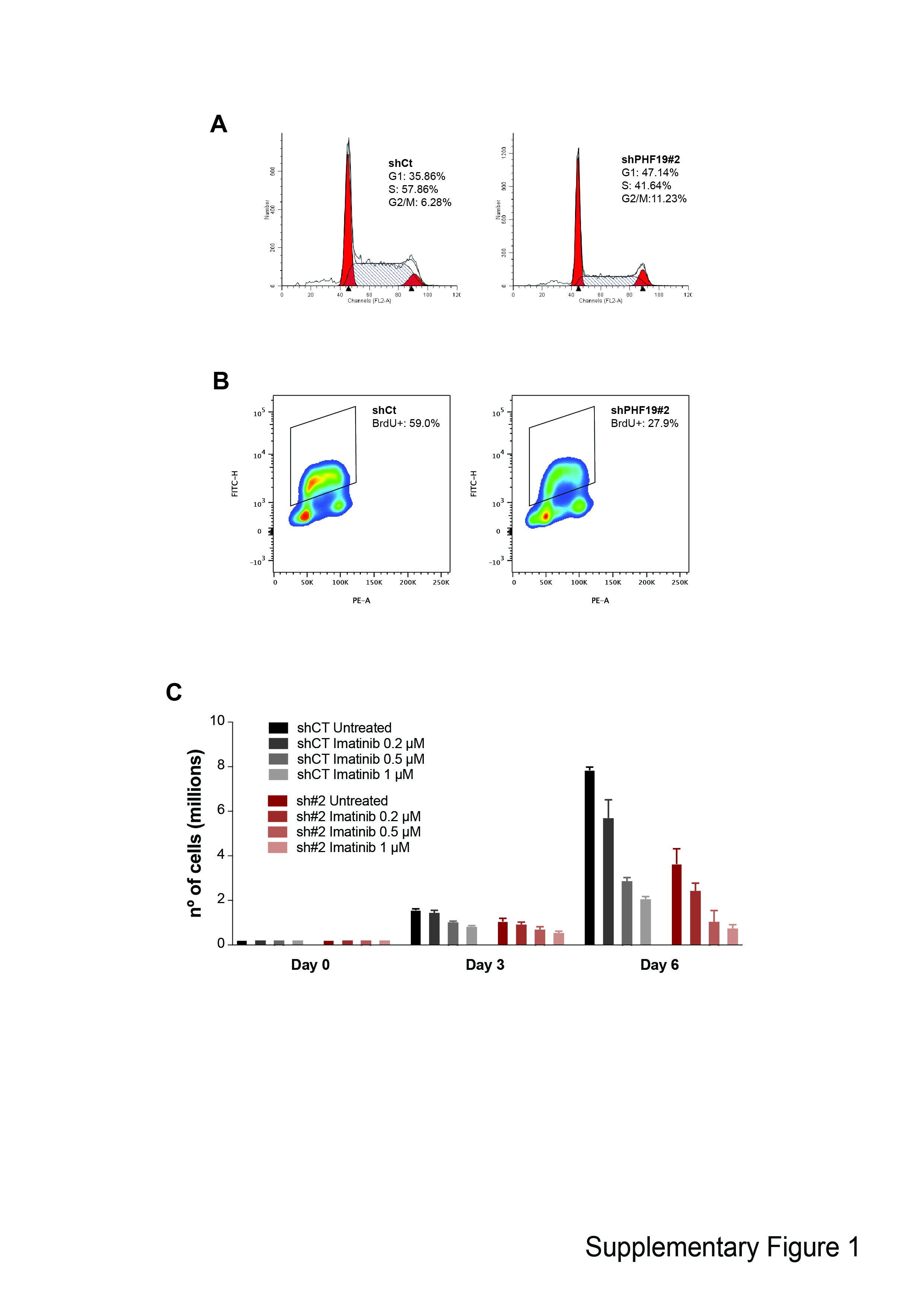

Supplement: Supplementary Figure 1 — (A) Representative analysis of cell cycle phases in shCT and shPHF19#2. (B) Representative analysis of BrdU incorporation analysis in shCT and shPHF19#2. (C) Cell growth of cells infected with an shCT and shPHF19#2 in the absence or presence of 10 nM Ara-C for 6 days. [file Image_1.jpg]

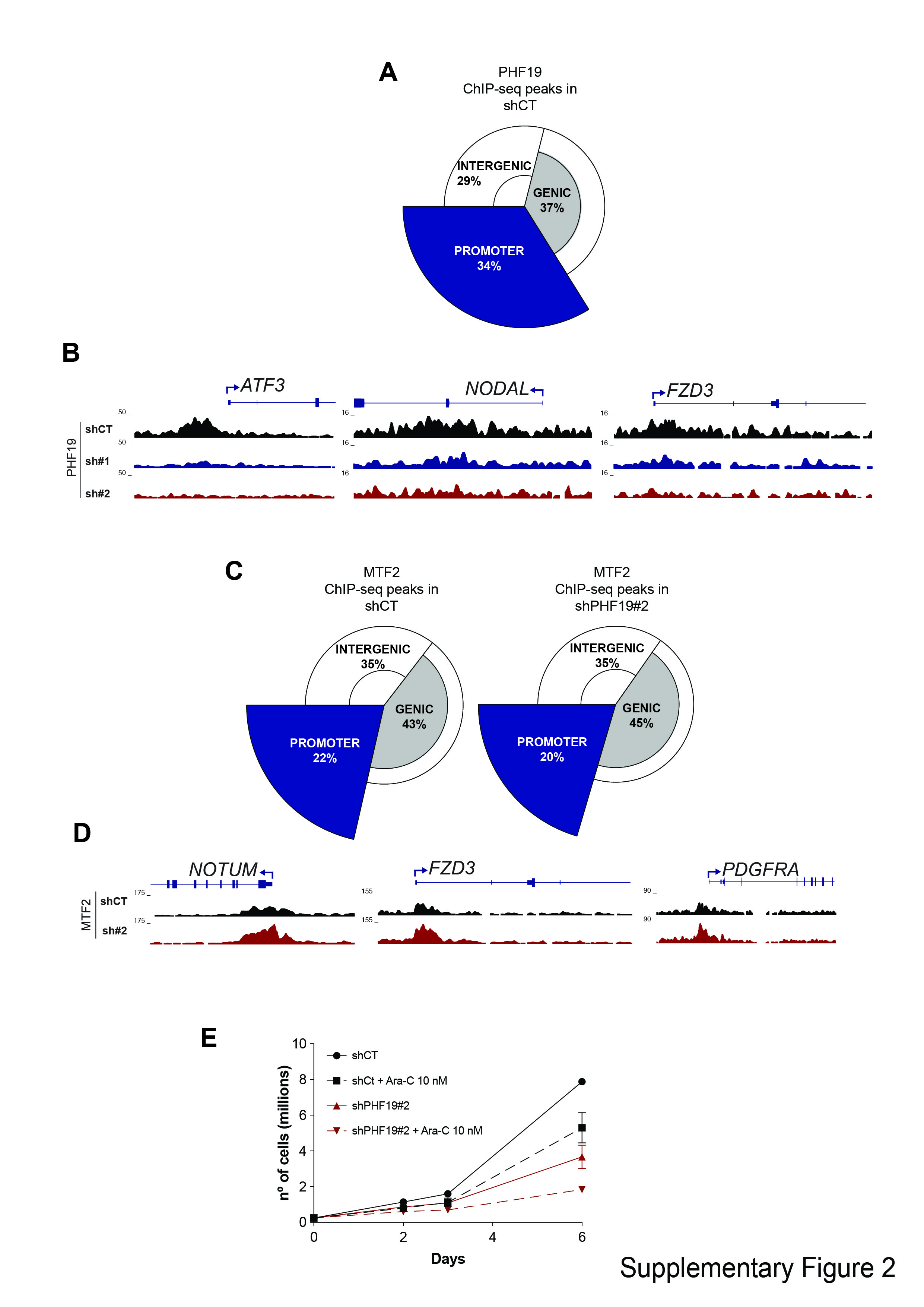

Supplement: Supplementary Figure 2 — (A) Genomic distribution of ChIP-seq peaks of PHF19. The spie-chart represents the genomic distribution of ChIP-seq peaks (outer circle) corrected by the whole-genome distribution of each gene genomic feature (indicated in the background circle distribution). (B) PHF19 ChIP-seq screenshots modified from UCSC genome browser of genes validated in Figure 3B. (C) Genomic distribution of ChIP-seq peaks of MTF2 (in shCT and shPHF19#2). The spie-chart represents the distribution of peaks corrected by the genome-wide distribution of each gene genomic feature (indicated in the background circle distribution). (D) MTF2 ChIP-seq screenshots modified from UCSC genome browser of genes validated in Figure 4B. (E) Accumulative growth of cells infected with an shCT and shPHF19#2 in the absence or presence of different doses of Imatinib for 6 days. [file Image_2.jpg]
